# Care recommendations for parturient and postpartum women and newborns during the COVID-19 pandemic: a scoping review^*^

**DOI:** 10.1590/1518-8345.4596.3359

**Published:** 2020-08-10

**Authors:** Victor Hugo Alves Mascarenhas, Adriana Caroci-Becker, Kelly Cristina Máxima Pereira Venâncio, Nayara Girardi Baraldi, Adelaide Caroci Durkin, Maria Luiza Gonzalez Riesco

**Affiliations:** 1 Universidade de São Paulo, Escola de Enfermagem, São Paulo, SP, Brazil.; 2 Scholarship holder at the Conselho Nacional de Desenvolvimento Científico e Tecnológico (CNPq), Brazil.; 3 Universidade de São Paulo, Escola de Artes, Ciências e Humanidades, São Paulo, SP, Brazil.; 4 Kettering College, Nursing, Kettering, OH, United States of America.; 5 Southern Adventist University, Graduate/DNP, Collegedale, TN, United States of America.

**Keywords:** Coronavirus Infections, Parturition, Postpartum Period, Infant, Newborn, Obstetrics, Neonatology, Infecciones por Coronavirus, Parto, Periodo Posparto, Recién Nacido, Obstetricia, Neonatología, Infecções por Coronavirus, Parto, Período Pós-Parto, Recém-Nascido, Obstetrícia, Neonatologia

## Abstract

**Objective:**

to map the current knowledge on recommendations for labor, childbirth, and newborn (NB) care in the context of the novel coronavirus.

**Method:**

scoping review of papers identified in databases, repositories, and reference lists of papers included in the study. Two researchers independently read the papers’ full texts, extracted and analyzed data, and synthesized content.

**Results:**

19 papers were included, the content of which was synthesized and organized into two conceptual categories: 1) Recommendations concerning childbirth with three subcategories – Indications to anticipate delivery, Route of delivery, and Preparation of the staff and birth room, and 2) Recommendations concerning postpartum care with four categories – Breastfeeding, NB care, Hospital discharge, and Care provided to NB at home.

**Conclusion:**

prevent the transmission of the virus in the pregnancy-postpartum cycle, assess whether there is a need to interrupt pregnancies, decrease the circulation of people, avoid skin-to-skin contact and water births, prefer epidural over general anesthesia, keep mothers who tested positive or are symptomatic isolated from NB, and encourage breastfeeding. Future studies are needed to address directed pushing, instrumental delivery, delayed umbilical cord clamping, and bathing NB immediately after birth.

## Abstract (es)

**Objetivo:**

mapear la producción de conocimientos sobre las recomendaciones para la atención a la mujer en el parto y postparto y al recién nacido (RN) ante la pandemia del nuevo coronavirus.

**Método:**

revisión sistemática exploratoria con selección de artículos en bases de datos, repositorios y listas de referencia de los estudios. Dos revisores independientes llevaron a cabo la lectura de los textos completos, la extracción de los datos, el análisis del material y la síntesis del contenido.

**Resultados:**

se incluyeron 19 artículos, que tuvieron el contenido resumido y presentado en dos categorías conceptuales: 1) Recomendaciones sobre la atención al parto, con tres subcategorías - Indicaciones para la anticipación del parto; Vía de nacimiento y Preparación del equipo y del medio ambiente para el nacimiento, y 2) Recomendaciones sobre la atención puerperal, con cuatro subcategorías - Lactancia materna; Cuidados con el RN; Alta hospitalaria y Cuidados domiciliarios con RN.

**Conclusión:**

en el ciclo gravídico-puerperal, se debe evitar la transmisión del virus, evaluar la necesidad de interrumpir la gestación, reducir la circulación de personas, evitar el contacto piel con piel y el parto en el agua, preferir la anestesia peridural a la anestesia general, mantener a la mujer aislada del RN y estimular la lactancia. Se necesitan estudios futuros sobre pujo dirigido, parto instrumental, pinzamiento tardío del cordón umbilical y baño inmediato del RN.

## Abstract (pt)

**Objetivo:**

mapear a produção de conhecimento sobre as recomendações para assistência ao parto, puerpério e cuidados com o recém-nascido em face da pandemia do novo coronavírus.

**Método:**

revisão de escopo com seleção dos artigos em bancos de dados, repositórios e em listas de referências dos estudos. Dois revisores independentes realizaram leitura dos textos completos, extração dos dados, análise do material e sintetização do conteúdo.

**Resultados:**

foram incluídos 19 artigos, que tiveram os conteúdos sintetizados e apresentados em duas categorias conceituais: 1) recomendações sobre a assistência ao parto, com três subcategorias – indicações para antecipação do parto; via de nascimento e preparação da equipe e do ambiente para o nascimento; 2) recomendações sobre a assistência puerperal, com quatro subcategorias – aleitamento materno; cuidados com o recém-nascido; alta hospitalar e cuidados domiciliares com recém-nascido.

**Conclusão:**

no ciclo gravídico-puerperal deve-se prevenir a transmissão do vírus, avaliar a necessidade da interrupção da gestação, reduzir a circulação de pessoas, evitar o contato pele a pele e o parto na água, preferir a anestesia peridural à anestesia geral, manter a mulher isolada do recém-nascido e estimular a amamentação. Estudos futuros sobre puxo dirigido, parto instrumental, clampeamento tardio do cordão umbilical e banho imediato do recém-nascido são necessários.

## Introduction

The World Health Organization (WHO) declared the human infection caused by the novel coronavirus Severe Acute Respiratory Syndrome-Coronavirus (SARS-CoV-2), named COVID-19”, a Public Health Emergency of International Concern”^(1)^.

This infection has been acknowledged as the most disturbing event since World War II and has put health systems worldwide under unprecedented stress. This context has led the entire population, health workers, and government officials to experience an atmosphere of fear and emotional stress, because of its severity and high mortality rates as well as the lack of sufficient services and equipment to meet the large demand of patients requiring hospitalization in Intensive Care Units and mechanical ventilators. On April 30th, 2020, infectious disease experts reported that this pandemic may last between 18 and 24 months and that everyone should be prepared for its resurgence after the first wave of contamination^(1-7)^.

To decrease infection or prevent the majority of the population from being infected at the same time, causing the health system to collapse, the WHO and Brazilian Ministry of Health have recommended social isolation, early detection, reporting, as well as investigation and appropriate case management^(1,8)^.

The novel coronavirus is transmitted through droplets and respiratory secretions of individuals infected by the disease or through contaminated objects; this virus can be also transmitted through contaminated feces^(5)^. Health workers are advised to observe contact and droplet precautions, according to the procedures performed. Hence, complete Personal Protective Equipment (PPE) has to be worn, such as disposable waterproof aprons or gown, goggles, head covers, gloves, and N95 masks or PFF2 respirators. Care is advised when removing PPE^(9)^.

Symptoms of SARS-CoV-2 may range from mild symptoms such as fever, runny nose, nasal congestion, dyspnea, malaise, myalgia, and loss of taste up to severe symptoms such as Severe Acute Respiratory Syndrome (SARS). Complications are most common, and more frequently lethal, among elderly individuals and those with comorbidities^(10-11)^. The Brazilian Ministry of Health classified women in the pregnancy-postpartum cycle and newborns (NB) to be risk groups^(11-13)^, considering that the clinical condition of these individuals may be aggravated by the infection due to low immunity and poor tolerance to hypoxia, which culminate in worse outcomes, compared to the population in general^(14-15)^.

The number of pregnant women and NB infected is much lower than that of the general population, however, pregnant and puerperal women are more vulnerable to COVID-19 and, when they become infected, the symptoms may be more severe. Transmission may occur from mother to NB in the postpartum and, the NB’s immunological system being still immature, they are believed to be more susceptible to SARS-CoV-2 infection. Hence, it is recommended to prevent infection of NB through contact with mothers, close family members, and health workers who are sick or carry the virus^(2,11,15)^.

One study conducted by Chinese researchers analyzed 2,143 cases of children younger than 18 years, 731 of whom were confirmed and 1,412 considered suggestive due to their clinical condition, imaging tests, and the fact they had been exposed to individuals with the virus. The study’s results show that children younger than one year had their clinical condition worsened^(15)^.

Decisions concerning the route of childbirth also need to take into account individual characteristics. As usual, C-sections should be indicated depending on the maternal and fetal conditions^(16)^. Note that even amidst this pandemic, good practices concerning labor, birth and postpartum care should continue among women who are not suggestive of being infected or had a confirmed diagnosis of COVID-19, as well as women who have recovered from the infection^(17)^.

There is little scientific evidence for the development of a protocol addressing the best treatment against the novel coronavirus. Therefore, this study is intended to map the body of knowledge acquired thus far concerning recommendations for childbirth, postpartum, and newborn care in the context of the novel coronavirus pandemic.

## Method

A scoping review was chosen because it permits a broad, comprehensive, and systematic exploration of the findings reported in the literature. This type of study is appropriate to collect the main recommendations for childbirth and postpartum, as it provides an overview of existing content without necessarily critiquing the methodological rigor of findings, an important factor at a time when information is still uncertain. According to Arksey and O’Malley, this method is implemented in five stages: (1) establishment of the research question; (2) identification of relevant studies; (3) selection and inclusion of studies; (4) data organization; and (5) collection, synthesis, and report of results^(18-19)^. These stages are individually described, as follows.

This review’s research question was: “What body of knowledge is available regarding the main recommendations for the care provided to parturient and postpartum women and newborns during the COVID-19 pandemic?” This question and the main elements for the search in this study were based on the PCC strategy (Population, Concept, and Context), which is the ideal method for a scoping review, according to the protocol released by the Joanna Briggs Institute (JBI)^(20)^. The population includes parturient and postpartum women and newborns. The main concept addressed here refers to the COVID-19 pandemic and the SARS-CoV-2 virus, and the contexts addressed are labor, childbirth, and postpartum in addition to the neonatal period.

The studies relevant in this review were identified through a search and selection process conducted in the following databases: Online Medical Literature Search and Analysis System - MEDLINE (via PubMed), Scopus, *Cumulative Index to Nursing and Allied Health Literature* (CINAHL), *Web of Science* (WoS), Latin-American and Caribbean Health Sciences Literature (LILACS) and Brazilian Nursing Database (BDENF). Descriptors that were appropriate to the databases (Medical Subject Headings - MeSH, CINAHL *Headings*, and Health Sciences Descriptors - DeCS) were used, along with keywords to broaden the search. Unpublished research was searched in the repository of the Brazilian Digital Library of Theses and Dissertations (BDTD) and the Theses and Dissertations Catalog provided by the Coordination for the Improvement of Higher Education Personnel (CAPES). Additionally, the reference lists of the main papers included in the study were verified to identify other pertinent papers.

The search strategy used with the selected terms was (“postpartum women” OR “postnatal women” OR “perinatal women” OR “pregnant women”) AND (“covid-19” OR “severe acute respiratory syndrome coronavirus 2” OR “severe acute respiratory syndrome coronavirus 2” OR “2019-nCoV” OR “SARS-CoV-2” OR “2019nCoV” OR “coronavirus”) AND (parturition OR “labor, obstetric” OR “labor stage, third” OR “labor stage, fourth” OR childbirth OR delivery OR postpartum OR puerperium OR “period, postpartum”).

The sources included met the following criteria: the full-texts of published or unpublished literature, written in English, Spanish or Portuguese, with no time frame. The search was conducted in April 2020 and included studies addressing maternal and neonatal variables during the childbirth, postpartum and neonatal periods in the context of the new coronavirus. Studies addressing the gestational period and prenatal care were excluded, along with papers that did not provide direct recommendations for the care provided to parturient and puerperal women and newborns.

The results of the search for papers were transferred to the bibliographic manager *Endnote Web*, an instrument that permits access by multiple researchers, the identification of duplicated papers, and the organization of the references in separate files, according to the database. To minimize selection bias, two independent researchers read and assessed the studies included. Disagreements were discussed until a consensus was obtained or with the help of a third reviewer.

Data were extracted and descriptively analyzed using an instrument the authors developed to characterize the manuscripts (year, country of origin, journal, authors), addressing methodological characteristics (study design, sample characteristics, summary of the method), and results (main recommendations to parturient and puerperal women and NB), and conclusions.

Afterward, thematic content analysis was performed to identify the key points, establish strengths, and the existing gaps in the literature. Finally, the results were revised regarding the main recommendations and practices performed thus far. The results were summarized in tables presenting the main findings and information is presented in the narrative form.

Due to the method’s specificity, there was no need to formally assess the methodological quality of the studies included. This review followed the PRISMA checklist to ensure methodological rigor and content of the report^(21)^.

## Results

**Figure 1:**
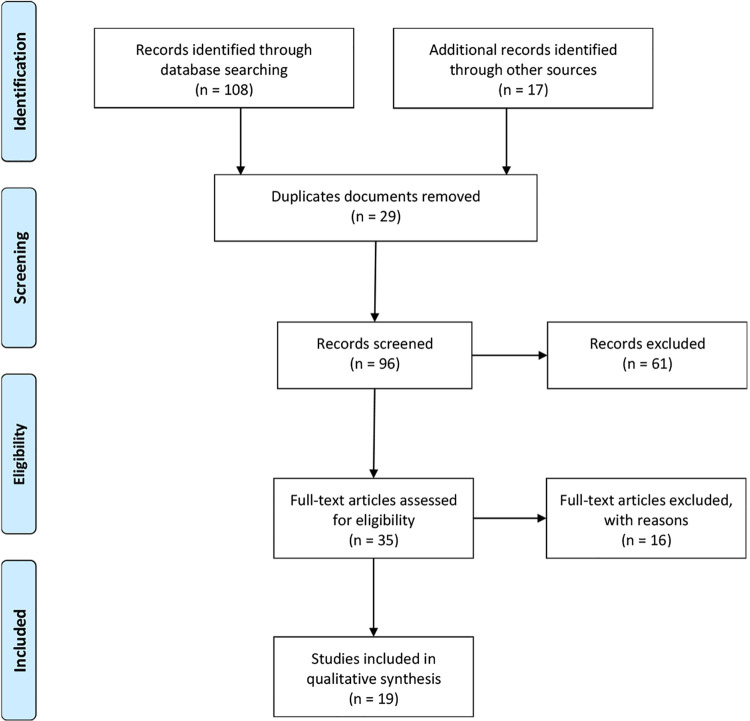
– Flowchart of the paper selection process, PRISMA-ScR. São Paulo, SP, Brazil, 2020

In total, 108 papers were identified in the databases, while 17 were identified in reference lists and unpublished research repositories. Of these, 29 appeared more than once and were removed. Hence, 96 papers were selected to read the titles and abstracts. Two researchers independently selected the papers, the full texts of which would be read. Then, 61 documents were excluded. The full texts of the remaining 35 eligible papers were read and 16 of these papers were excluded because they did not address the research question or did not provide significant recommendations to be included in this review. Hence, 19 met the inclusion criteria and remained in the final sample (Figure 1).

Most papers included in this review were studies conducted in China (n=13), the country where the epidemic of the novel coronavirus started. Even after the pandemic had been reported, other countries also experienced the growing presence of the novel coronavirus, however, scientific research was still incipient in these countries, with only two papers conducted in the United States and two in Singapore.

**Figure 2:**
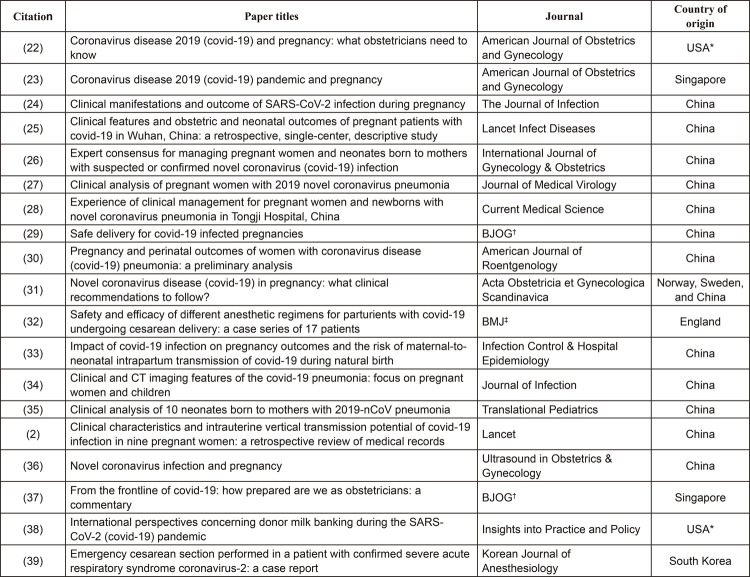
– Records included by scoping review according to title, journal, and country of origin. São Paulo, SP, Brazil, 2020 *USA = United States of America; ^†^BJOG = British Journal of Obstetrics and Gynaecology; ^‡^BMJ = British Medical Journal

Even though there was an attempt to decrease the language bias, all the studies were published in English, despite the origin of the publications. Due to the current nature of the topic addressed, all studies dated from 2020 and comprised the first scientific documents available thus far. The journals comprised different medical specialties, not limited to gynecology and obstetrics, showing the importance of various fields addressing this subject. The characteristics of the studies included here are detailed in Figure 2.

Regarding the methodological designs of the papers included in this review, these include six retrospective descriptive studies, five reviews, four opinion papers, three case studies, and one experience report.

To facilitate the presentation of information collected from the manuscripts, after reading and analyzing recommendations, the content was grouped into two general conceptual categories: 1) Recommendations regarding care provided to childbirth in the context of the SARS-CoV-2 infection, which consisted of three subcategories (Indications to anticipate childbirth; Route of childbirth; and Preparation of the staff and childbirth room), and 2) Recommendations for postpartum care provided in the context of the SARS-CoV-2 infection, which comprised four subcategories (Breastfeeding; Newborn care; Hospital discharge; and Care provided to NB at home).

**Figure 3:**
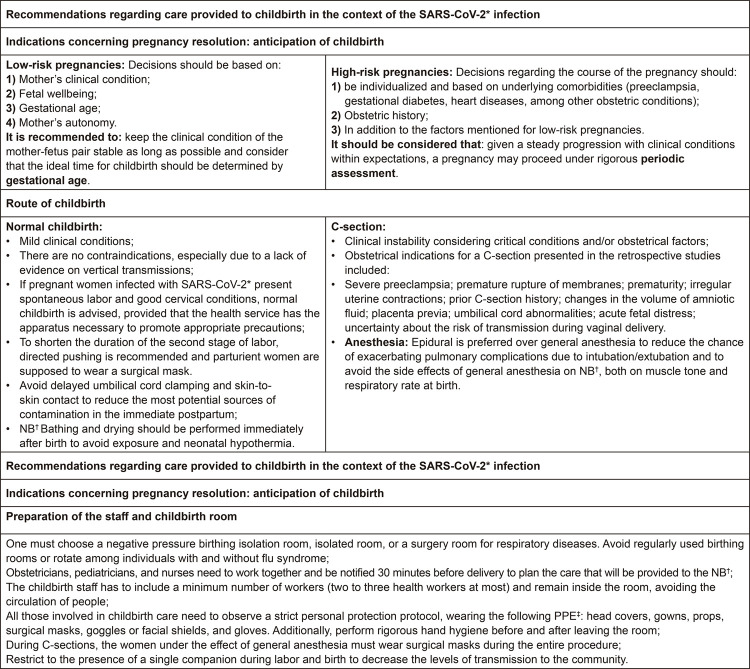
– Main recommendations for childbirth care in the context of SARS-CoV-2 infection. São Paulo, SP, Brazil, 2020 *SARS-CoV-2 = Severe Acute Respiratory Syndrome-Coronavirus; ^†^NB = Newborn;^‡^PPE = Personal Protective Equipment

**Figure 4:**
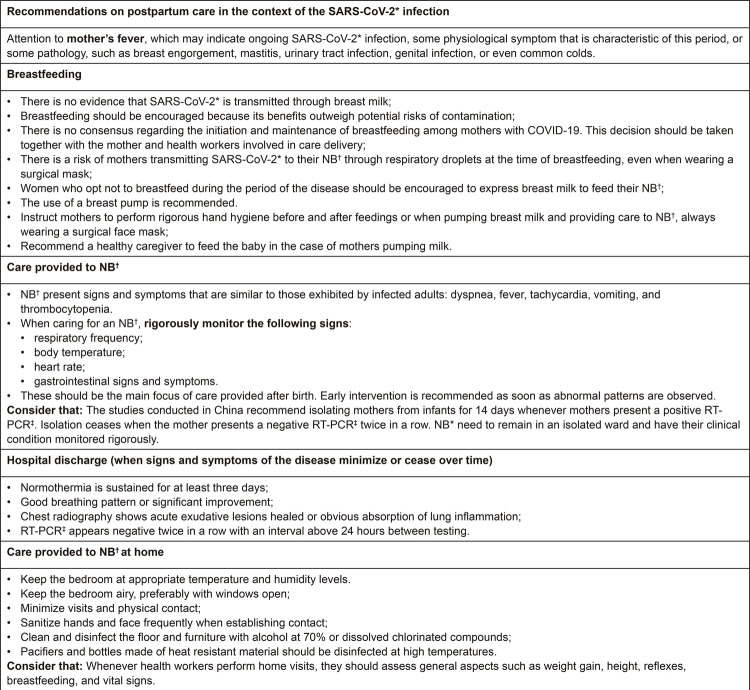
– Main recommendations for postpartum and newborn care in the context of SARS-CoV-2 infection. São Paulo, SP, Brazil, 2020 *SARS-CoV-2 = Severe Acute Respiratory Syndrome-Coronavirus; ^†^NB = Newborn; ^‡^RT-PCR = Reverse Transcription Polymerase Chain Reaction

The contents of these categories are described and represented in Figures 3 and 4:

## Discussion

This scoping review allowed mapping the body of knowledge currently available on how to deal with COVID-19 during the pregnancy-postpartum cycle and the main recommendations regarding labor, childbirth, postpartum, and breastfeeding. This topic should be a priority and has gained attention after a group of Brazilian researchers developed a survey in the field of women’s health. This document reports that five deaths caused by COVID-19 occurred in a context of 1,947 deaths while, in Iran, two maternal deaths were reported in a total of 3,800 deaths. Little has been mentioned about maternal deaths in the context of the pandemic in European countries or even in the remaining countries in the Americas. Hence, attention should be drawn to maternal deaths caused by the novel coronavirus^(40)^.

Even with limited results concerning how the SARS-CoV-2 infection affects parturient and postpartum women, greater deterioration of the health condition of mothers has been observed in Brazil. Therefore, the Brazilian Ministry of Health included pregnant women, those who gave birth recently, and those who experienced an abortion or fetal death in the group of risk and provided specific guidelines^(41-43)^. One recent study^(44)^ addressing 53 Swedish women aged between 20 and 45 years old identified that 13 of them were in the pregnant-postpartum cycle. The results show that these pregnant and postpartum women needed intensive care and seven of them required mechanical ventilation. Even with limitations due to the sample size, the researchers state that pregnant women more frequently demand intensive care compared to non-pregnant women. Therefore, greater care is recommended with this group given the potential of positive cases for SARS-CoV-2 to become more severe.

In this context, a suggestive or confirmed diagnosis of COVID-19 has promoted changes in the context of childbirth and obstetric care that is provided to women with the infection. There was a need for a rigorous evaluation of pregnancy and fetal status due to the tendency to fetal growth restriction and greater chances of prematurity^(31,41,45)^.

Decisions regarding the course of pregnancy or its resolution should take into account: the mother’s clinical condition, fetal wellbeing, gestational age, and the mother’s autonomy. As long as the clinical condition of both mother and fetus is ensured, the ideal time for delivery should be determined by gestational age^(22,26,29,31)^. If, however, the safety of either mother or fetus is compromised due to SARS or there is no satisfactory response to the therapy implemented against COVID-19, or yet, in the presence of severe pneumonia or critical condition, a premature delivery should be considered to safeguard mother and infant, as an anticipated birth decreases fetal hypoxemia and the maternal condition is expected to improve^(28-29,31)^.

In some cases, the interruption of pregnancy should be considered, even before fetal viability is achieved, seeking to improve the mother’s clinical condition, considering that there is evidence showing rapid recovery of maternal oxygenation after the procedure^28-29,31^). Any decision by the health staff should consider the mother’s and family’s principles and desires, without disregarding ethical principles^(31)^. These precepts are endorsed by the International Confederation of Midwives, which reinforce the need of women and fetuses to be treated with dignity, compassion, and respect^(46)^.

Regarding the birth route, studies^(23,28,32,47)^ show that there is no contraindication for vaginal deliveries if clinical and obstetrical assessments are favorable. Greater surveillance of both mother and fetus is recommended through cardiotocography or intermittent auscultation in a short period. Water births are contraindicated due to the possibility for the mother’s fecal elimination and water contamination, and consequently, greater risk of contamination of NB^(5)^. C-sections should be indicated when there is clinical or obstetrical instability in addition to changes in fetal vitality^(28,32,35,38,47)^. These recommendations are corroborated by obstetricians in Wuhan, China, who state that C-sections indicated for women with the infection should be flexible, as the objective is to decrease the length of hospitalization of mothers to minimize cross-infection and avoid physical exertion, which is characteristic of normal deliveries^(29)^.

The staff providing childbirth care must be informed about a suspected or confirmed diagnosis of COVID-19 to prepare for and implement biosafety measures, as well as to grant priority for these deliveries to occur in labor, childbirth and postpartum rooms reserved for those with a suspected or confirmed diagnosis of the SARS-CoV-2 infection^(26,28-29)^. At the time of delivery, a minimum team of essential workers should be considered; hygiene measures and protective equipment guidelines should be rigorously observed; parturient women should wear surgical or N95 masks; and only one companion should be recommended during labor, childbirth and postpartum^(23,26,29,34-45,48)^. This companion should be someone who lives with the mother, does not belong to a risk group, does not present any signs and symptoms of the flu; and wears a mask during his/her entire permanence in the service^(45)^.

Recommendations to hasten the expulsive period by promoting directed pushing or instrumental delivery demand caution because there is no consensus and this procedure may increase the risk of exposure, as it decreases the efficacy of face masks in preventing the propagation of particles^(36)^. Furthermore, there is a lack of evidence on whether the abbreviation of labor improves maternal and fetal outcomes. Epidural analgesia is preferred in the case of mothers wishing pharmacological analgesia during childbirth^(48)^. This option should also be offered in the case of C-sections, considering that general anesthesia may cause pulmonary complications related to intubation/extubation, in addition to side effects for NB^(29,32,39)^.

It is important to highlight one review addressing the guidelines concerning pregnant women and NB in the context of COVID-19, reporting that there is no consensus among the practices adopted in the different countries and that these recommendations may differ between countries, as each can give priority to follow local health government agencies or international organizations^(48)^.

The Chinese studies included in this review recommend isolating the mother-infant pair after birth, as this procedure is believed to decrease NB contamination by SARS-CoV-2. For this reason, these studies recommend delayed umbilical cord clamping and skin-to-skin contact to be avoided. Therefore, NB should be placed in a warm crib in an environment different from that of mothers^(23,26,31,37)^. One review^(48)^, a guide providing guidelines for pregnancy, labor and childbirth^(5)^, and a technical note from the Brazilian Ministry of Health^(41)^ report, however, that umbilical cord clamping among asymptomatic women, and even among women with symptoms, does not need to be immediate. Because there is no evidence of vertical transmission, optimal timing for umbilical cord clamping may be chosen instead of immediate clamping.

It is important to consider that the pandemic demands that health workers and health services exercise good judgment and assess each case individually, encouraging and adopting good practices to promote a positive experience for mothers. The desire and clinical conditions of women should be taken into account, and physical structures should be adapted, observing biosafety maneuvers necessary to decrease the chances of virus transmission^(45-46,49)^.

Vigilant attention should be intensified in the postpartum period to monitor the mother’s signs and symptoms and detect any worsening of the mothers’ health conditions early^(43)^. Even though hyperthermia is the symptom most frequently reported in the SARS-CoV-2 infection, a differential diagnosis should be made, considering that other pathologies may be associated, while other specific signs and symptoms of the respiratory infection caused by COVID-19 should be also investigated^(28)^.

The hospital discharge of mothers depends on their health conditions. Chinese studies recommend waiting for the minimization or complete disappearance of signs or symptoms over time. Therefore, postpartum women are expected to present: sustained normothermia for at least three days; improved respiratory and clinical standards; involution of acute exudative lesions or lung inflammation; and to test negatively in Reverse Transcription Polymerase Chain Reaction (RT-PCR) twice in a row, with intervals greater than 24 hours between tests^(23,28)^. The discharge of mothers in situations of social vulnerability, comorbidities or pregnancy complications, or with a COVID-19 diagnosis, should occur after health workers ensure that these women have established a systematic monitoring flow within the primary health care network and have access to specialized care in case their condition aggravates^(42,50)^. In such a context, the state of São Paulo, Brazil establishes that pregnant, parturient and postpartum women are referred via system by *Central de Regulação de Ofertas de Serviços de Saúde* (CROSS) [Central Regulation for the Supply of Health Services], granting priority to those in this phase of the lifecycle, considering the need for early detection and severity in this risk group^(51)^.

Regarding breastfeeding, the postpartum women in China are advised against breastfeeding based on previous experiences with SARS and also because the antiviral drug lopinavir/ritonavir, which is chosen for the treatment against COVID-19, is excreted through breast milk^(27-28,37)^. Evidence, however, shows that there is no virus in human breast milk. Therefore, breastfeeding should be encouraged, especially when we consider the benefits of immunization for the NB^(2,23,26,33,38,46,52)^.

Currently, the greatest concern with breastfeeding lies in the possibility of infants being contaminated by the mothers’ respiratory droplets^(33,38)^. To decrease this risk, mothers are recommended to wear surgical masks during breastfeeding to protect against coughing or sneezing, in addition to constantly performing proper hand hygiene. If mothers opt not to breastfeed while the symptoms remain, health workers should encourage them to pump breast milk and allow another caregiver, who lives in the same home, to feed the infant^(23,28,38,52)^.

Bottles, dosing spoons, or glasses used to feed infants with breast milk have to be sterilized^(52)^. A multi-center retrospective study conducted in Italy addressing 42 women, recommend physicians and midwives, working in regions with high contamination rates by COVID-19, to encourage women to wear face masks, both during labor and childbirth and when providing care to infants and breastfeeding, aiming to decrease transmission to NB, considering that individuals with the infection may be asymptomatic^(47)^.

As for the NB in general, neonatal variables immediately after birth were satisfactory in terms of weight, height, and Apgar indexes, while no asphyxia or neonatal death was reported^(2,23-25,27,30,33-34)^. Even though infection due to the COVID-19 is less frequent in this population, attention should be paid to the risk of infection with greater severity among NB and children under three months of age^(53)^. Therefore, one should monitor for signs and symptoms, such as dyspnea and tachypnea, hyperthermia or hypothermia, tachycardia, emesis, gastrointestinal symptoms, and thrombocytopenia. In the case of infection, early intervention and differential diagnosis should apply^(25,31,33,35,45)^.

After hospital discharge, the routine and care provided at home are important to prevent the infant and other family members from becoming infected with SARS-CoV-2. Hence, mothers are recommended to be isolated until the symptoms disappear or present negative RT-PCR; keep at least two-meter distance from NB when not breastfeeding or providing childcare; keep bedrooms airy and with appropriate temperature and humidity levels; minimize or completely disallow visits and physical contact. It is also important to clean and disinfect the floor and furniture with alcohol or dissolved chlorinated compounds^(28)^. The Brazilian Ministry of Health reports an acceptable distance of one meter between mother and NB^(42)^.

The general health aspects of mother and infant should be assessed during home visits, childcare, and postpartum consultations. Also, checks should include anthropometric measures, weight gain, reflexes, breastfeeding, contraceptive methods, how the mother is adapting to the postpartum period, and vital signs^(28,45)^. Additionally, reproductive planning is indicated to ensure the safety of contraceptive methods. It should even be intensified, considering that there is a lack of strong evidence concerning vertical transmission, premature labor, and restricted intrauterine growth. It is also noteworthy that parturient and postpartum women and newborns have been included in vulnerable groups. The choice of the contraceptive should consider the desires of women and their families, although the intrauterine device is an option that can be offered in the immediate postpartum period^(40,42,46)^.

Finally, most of the studies included in this scoping review were conducted in China because it is where the novel coronavirus originated. Retrospective studies were most frequent, followed by reviews, opinion papers, and experience reports, a fact that denotes poor scientific evidence. Nonetheless, this scoping review contributes to scientific advancement as it shows, through the mapping of knowledge available thus far, the main clinical, obstetrical, and neonatal recommendations to deal with the COVID-19 among vulnerable groups, the population addressed here. Additionally, these recommendations can improve reflections on clinical practices. In the scientific field, this study encourages future studies to involve this topic and method, considering the high flow of scientific knowledge regarding the novel coronavirus and the COVID-19.

The potential limitations of this scoping review include the fact that most studies were conducted in a single country (China), which may increase information bias, and the fact that other socio-cultural contexts are not contextualized. Note also that there is a lack of controlled clinical trials or observational studies. The sudden onset of the pandemic and intense flow of information hinders the availability of strong and/or sound recommendations. The decision to select studies written in three different languages limited the findings, as most of the scientific knowledge has been produced in China and was not available in the languages selected for this review.

## Conclusion

Women in the pregnancy-postpartum cycle and NB are more vulnerable to complications when infected by the novel coronavirus. Thus, it is important to acquire knowledge on the main recommendations regarding the care that is provided to women during childbirth, postpartum, and also childcare.

Due to the recent emergence of the COVID-19, there is not sufficient scientific evidence to provide precise guidelines and protocols to fight the disease. Nonetheless, prior knowledge from different health fields and the results reported by the papers available enabled mapping the various practices that can be recommended in obstetrical and neonatal care, as follows: companions and health workers are supposed to prevent the transmission of the virus to NB by adopting isolation measures and contact, droplets and/or aerosols precautions; keep strict hand hygiene; health workers, mothers, and companions must wear PPE when providing care to NB; consider when a pregnancy should be interrupted or anticipated; decrease the circulation and number of people involved in the care provided to women and NB. Skin-to-skin contact during childbirth and water births is not recommended. The route of childbirth should be chosen following conventional obstetrical indications and the women’s clinical conditions. If anesthesia is indicated, the epidural is preferred over general anesthesia.

In some cases, women are advised to keep a distance from NB, though each case should be considered individually, taking into account the desires of women and their companions. Breastfeeding is recommended even among women infected with COVID-19, provided that they wear surgical masks and observe hand hygiene. When breastfeeding is impossible, mothers are recommended to express or pump breast milk. Vital signs should be rigorously assessed and symptoms of this infection identified during childcare consultations. Also, support such as medication, oxygen, and guidance concerning sleeping, resting, hydration, feeding should be provided in case of worsened symptoms, along with multidisciplinary care.

Further studies with greater methodological rigor are needed to resolve controversies regarding directed pushing, instrumental delivery, delayed umbilical cord clamping and bathing NB immediately after birth. Because this content is new, some recommendations may change as new knowledge and guidelines emerge in each country.

## References

[1] Word Health Organization (2020). Novel Coronavirus (2019-nCoV).

[2] Chen H, Guo J, Wang C, Luo F, Yu X, Zhang W (2020). Clinical characteristics and intrauterine vertical transmission
potential of covid-19 infection in nine pregnant women: a retrospective
review of medical records. Lancet.

[3] Secretaria de Estado da Saúde (BR), Coordenadoria de Controle de Doenças (2020). Plano de Contingência do Estado de São Paulo para Infecção Humana pelo
novo Coronavírus – 2019 nCOV.

[4] Sociedade Brasileira de Pediatria (BR) (2020). Recomendações para cuidados e assistência ao recém-nascido com suspeita
ou diagnóstico de COVID-19.

[5] Royal College of Obstetricians and Gynaecologists (UK) (2020). The Royal College of Midwifes. Coronavirus (COVID-19) Infection in
pregnancy. Information for healthcare professionals.

[6] Kamps BS, Hoffmann C (2020). COVID Reference ENG 2020.3.

[7] Center for Infectious Disease Research and Policy (2020). COVID-19: The CIDRAP viewpoint.

[8] Ministério da Saúde (BR), Secretaria de Ciência, Tecnologia, Inovação e Insumos Estratégicos
em Saúde (2020). Diretrizes para diagnóstico e tratamento da Covid-19.

[9] Sociedade de Pediatria de São Paulo Recomendações para assistência ao recém-nascido na sala de parto de mãe
com COVID-19 suspeita ou confirmada.

[10] Rodriguez-Morales AJ, Cardona-Ospina JA, Gutiérrez-Ocampo E, Holguin-Rivera Y, Escalera-Antezana JP, Alvarado-Arnez LE (2020). Clinical, laboratory and imaging features of COVID-19: a
systematic review and meta-analysis. Travel Med Infect Dis.

[11] Ministério da Saúde (BR), Secretaria de Atenção Primária à Saúde (2020). Nota Técnica nº 10/2020-COCAM/CGCIVI/DAPES/SAPS/MS. Atenção à saúde do
recém-nascido no contexto da infecção pelo novo coronavírus
(SARS-CoV-2).

[12] Ministério da Saúde, Secretaria de Atenção Primária à Saúde (2020). Nota Técnica nº 13/2020-COSMU/CGCIVI/DAPES/SAPS/MS. Infecção COVID-19 e
os riscos às mulheres no ciclo gravídico-puerperal.

[13] Ministério da Saúde, Secretaria de Atenção Primária à Saúde (2020). Protocolo de manejo clínico do coronavírus (covid-19) na atenção
primária à saúde.

[14] World Health Organization (2013). Essential nutrition actions: improving maternal, newborn, infant and
young child health and nutrition.

[15] Dong Y, Mo X, Hu Y, Qi X, Jiang F, Jiang Z (2020). Epidemiological characteristics of 2,143 pediatric patients with
2019 coronavirus disease in China. Pediatrics.

[16] World Health Organization (2020). Q&A: COVID-19 and pregnancy and childbirth.

[17] Estado de São Paulo (BR) (2020). Decreto no 64.864, de 16 de março de 2020. Dispõe sobre a adoção
de medidas adicionais, de caráter temporário e emergencial, de prevenção de
contágio pelo COVID-19 (Novo Coronavírus), e dá providências
correlatas. Diário Oficial do Estado de São Paulo.

[18] Peters MDJ, Godfrey CM, Khalil H, McInerney P, Parker D, Soares CB (2015). Guidance for conducting systematic scoping
reviews. Int J Evid Based Healthc.

[19] Arksey H, O’Malley L (2005). Scoping studies: towards a methodological
framework. Int J Soc Res Methodol.

[20] The Joanna Briggs Institute (2015). Joanna Briggs Institute Reviewers’ Manual: 2015 edition/
Supplement.

[21] Tricco AC, Lillie E, Zarin W, O’Brien KK, Colquhoun H, Levac D (2018). PRISMA Extension for scoping reviews (PRISMA-ScR): checklist and
explanation. Ann Intern Med.

[22] Rasmussen SA, Smulian JC, Lednicky JA, Wen TS, Jamieson DJ (2020). Coronavirus disease 2019 (COVID-19) and pregnancy: what
obstetricians need to know. Am J Obstet Gynecol.

[23] Dashraath P, Jing Lin JW, Mei Xian KL, Li Min L, Sarah L, Biswas A (2020). Coronavirus disease 2019 (COVID-19) pandemic and
pregnancy. Am J Obstet Gynecol.

[24] Liu Y, Chen H, Tang K, Guo Y (2020). Clinical manifestations and outcome of SARS-CoV-2 infection
during pregnancy. J Infect. Dis.

[25] Yu N, Li W, Kang Q, Xiong Z, Wang S, Lin X (2020). Clinical features and obstetric and neonatal outcomes of pregnant
patients with COVID-19 in Wuhan, China: a retrospective, single-centre,
descriptive study. Lancet Infect Dis.

[26] Chen D, Yang H, Cao Y, Cheng W, Duan T, Fan C (2020). Expert consensus for managing pregnant women and neonates born to
mothers with suspected or confirmed novel coronavirus (COVID-19)
infection. Int J Gynaecol Obstet.

[27] Chen S, Liao E, Shao Y (2020). Clinical analysis of pregnant women with 2019 novel coronavirus
pneumonia. J Med Virol.

[28] Wang SS, Zhou X, Lin XG, Liu YY, Wu JL, Sharifu LM (2020). Experience of clinical management for pregnant women and newborns
with novel coronavirus pneumonia in Tongji Hospital, China. Curr Med Sci.

[29] Qi H, Luo X, Zheng Y, Zhang H, Li J, Zou L (2020). Safe delivery for COVID-19 infected pregnancies. BJOG.

[30] Liu D, Li L, Wu X, Zheng D, Wang J, Yang L (2020). Pregnancy and perinatal outcomes of women with coronavirus
disease (COVID-19) pneumonia: a preliminary analysis. AJR Am J Roentgenol.

[31] Liang H, Acharya G (2020). Novel corona virus disease (COVID-19) in pregnancy: what clinical
recommendations to follow?. Acta Obstet Gynecol Scand.

[32] Chen R, Zhang Y, Huang L, Cheng BH, Xia ZY, Meng QT (2020). Safety and efficacy of different anesthetic regimens for
parturients with COVID-19 undergoing cesarean delivery: a case series of 17
patients. Can J Anaesth.

[33] Suliman K, Liangyu P, Rabeea S, Ghulam N, Nawsherwan MX, Jianbo Liu GH (2020). Impact of COVID-19 infection on pregnancy outcomes and the risk
of maternal-to-neonatal intrapartum transmission of COVID-19 during natural
birth. Infect Control Hosp Epidemiol.

[34] Liu H, Liu F, Li J, Zhang T, Wang D, Lan W (2020). Clinical and CT imaging features of the COVID-19 pneumonia: focus
on pregnant women and children. J Infect.

[35] Zhu H, Wang L, Fang C, Peng S, Zhang L, Chang G (2020). Clinical analysis of 10 neonates born to mothers with 2019-nCoV
pneumonia. Transl Pediatr.

[36] Yang H, Wang C, Poon LC (2020). Novel coronavirus infection and pregnancy. Ultrasound Obstet Gynecol.

[37] Chua MSQ, Lee JCS, Sulaiman S, Tan HK (2020). From the frontline of COVID-19 - how prepared are we as
obstetricians: a commentary. BJOG.

[38] Marinelli KA (2020). International perspectives concerning donor milk banking during
the SARS-CoV-2 (COVID-19) pandemic. J Hum Lact.

[39] Lee DH, Lee J, Kim E, Woo K, Park HY, An J (2020). Emergency cesarean section on severe acute respiratory syndrome
coronavirus 2 (SARS- CoV-2) confirmed patient. Korean J Anesthesiol.

[40] Amorim MMR, Takemoto MLS, Fonseca EB (2020). Maternal deaths with Covid-19: a different outcome from mid to
low resource countries?. Am J Obstet Gynecol.

[41] Estado de São Paulo (BR), Coordenadoria de Controle de Doenças (CCD- SP) (2020). Nota Técnica nº 03: Manejo do ciclo gravídico-puerperal e lactação -
Covid-19.

[42] Ministério da Saúde (BR), Secretaria de Atenção Primária à Saúde, Departamento de Ações Programáticas Estratégicas, Coordenação-Geral de Ciclos da Vida, Coordenação de Saúde das Mulheres (2020). Nota Técnica Nº 13/2020 - Recomendação acerca da atenção puerperal, alta
segura e contracepção durante a pandemia da COVID-19.

[43] Secretaria de Atenção Especializada à Saúde (BR) (2020). Protocolo de manejo clínico da Covid-19 na atenção
especializada.

[44] Collin J, Byström E, Carnahan A, Ahrne M (2020). Pregnant and postpartum women with SARS-CoV-2 infection in
intensive care in Sweden. Acta Obstet Gynecol Scand.

[45] Brigagão JIM, Caroci-Becker A, Baraldi NG, Feliciano RG, Venâncio KCMP, Mascarenhas VHA (2020). Recomendações e estratégias para o enfrentamento da Covid-19 durante a
gestação, o parto, o pós-parto e nos cuidados com o recém-nascido.

[46] International Confederation of Midwives (2020). Women’s rights in childbirth must be upheld during the coronavirus
pandemic.

[47] Ferrazzi E, Frigerio L, Savasi V, Vergani P, Prefumo F, Barresi S (2020). Vaginal delivery in SARS-CoV-2 infected pregnant women in
Northern Italy: a retrospective analysis. BJOG.

[48] Cochrane pregnancy and childbirth (2020). COVID-19 review of national clinical practice guidelines for key
questions relating to the care of pregnant women and their babies.

[49] World Health Organization (2018). WHO recommendations: intrapartum care for a positive childbirth
experience.

[50] Ministério da Saúde (BR), Secretaria de Atenção Primária à Saúde (2020). Nota Técnica nº 12/2020: Infecção COVID-19 e os riscos às mulheres no
ciclo gravídico-puerperal.

[51] Estado de São Paulo, Coordenadoria de Controle de Doenças (2020). Manejo ciclo gravídico puerperal - Covid 19: referência e contra
referência para a Região Metropolitana de São Paulo. Diário Oficial do Estado de São Paulo.

[52] Ministério da Saúde (BR) (2020). Nota Técnica nº 7/2020 - Covid-19 e amamentação.

[53] Vilelas JMS (2020). O novo coronavírus e o risco para a saúde das
crianças. Rev. Latino-Am. Enfermagem.

